# Nephrotic Syndrome: Oedema Formation and Its Treatment With Diuretics

**DOI:** 10.3389/fphys.2018.01868

**Published:** 2019-01-15

**Authors:** Sanjana Gupta, Ruth J. Pepper, Neil Ashman, Stephen B. Walsh

**Affiliations:** ^1^UCL Centre for Nephrology, University College London, London, United Kingdom; ^2^Renal Unit, The Royal London Hospital, Bart’s Health NHS Trust, London, United Kingdom

**Keywords:** nephrotic syndrome, diuretics, oedema, amiloride, epithelial sodium channel

## Abstract

Oedema is a defining element of the nephrotic syndrome. Its’ management varies considerably between clinicians, with no national or international clinical guidelines, and hence variable outcomes. Oedema may have serious sequelae such as immobility, skin breakdown and local or systemic infection. Treatment of nephrotic oedema is often of limited efficacy, with frequent side-effects and interactions with other pharmacotherapy. Here, we describe the current paradigms of oedema in nephrosis, including insights into emerging mechanisms such as the role of the abnormal activation of the epithelial sodium channel in the collecting duct. We then discuss the physiological basis for traditional and novel therapies for the treatment of nephrotic oedema. Despite being the cardinal symptom of nephrosis, few clinical studies guide clinicians to the rational use of therapy. This is reflected in the scarcity of publications in this field; it is time to undertake new clinical trials to direct clinical practice.

## Introduction

Interstitial oedema is present in all individuals with nephrotic syndrome and can be profound, accounting for as much as an additional 30% to an individual’s total body weight (Doucet et al., 2007). Oedema is one of the four defining features of the nephrotic syndrome and is the symptom most commonly requiring intervention (Crew et al., 2004). The other three defining features of nephrotic syndrome are hypoalbuminemia, hyperlipidemia, and proteinuria.

The prevalence of nephrotic syndrome in a small historical retrospective study from 1996 in the United States reported that 60% of all renal biopsies were performed to establish a diagnosis after this presentation (Korbet et al., 1996). It is plausible that this number has not significantly changed. The incidence of primary glomerulonephritides causing the nephrotic syndrome between 1980 to 2010 (limited to the three most common histological findings: minimal change disease, membranous nephropathy and focal segmental glomerular sclerosis) in adults was 2.6 per 100,000 per year (McGrogan et al., 2011).

The burden of symptomatic disease is high, mainly with oedema. Parents of oedematous children report increased anxiety and multiple emergency hospital visits (Beanlands et al., 2017). Peripheral oedema may be uncomfortable, leading to functional restraint, restricted leg movement, and impaired mobility (Doucet et al., 2007). Oedema may also cause increased skin tension, resulting in blistering, skin breakage and exudate extrusion, offering an encouraging environment for bacterial infection. The risk of infection is exacerbated by ‘nephrotic immunodeficiency’ caused by the urinary loss of immunoglobulins (Ogi et al., 1994) and T-cell transformation dysfunction (Fodor et al., 1982). Further, asymptomatic pulmonary congestion can be present in active nephrosis (Marino et al., 2016) and there is a strong association of cardiovascular risk in an overloaded chronic kidney disease cohort (Hung et al., 2014).

The two main contributors to oedema are the urinary loss of albumin and excessive sodium. Hence, diuretics are used due to the known tubular effects on sodium and water reabsorption. Before we can target and tailor treatment, we first need to understand the underlying physiology of oedema formation.

## Oedema Formation

There are two paradigms of oedema formation in nephrosis: the so-called under-fill and over-fill models; it is thought that these can be present in the same individual at different times over the course of their disease (Perico and Remuzzi, 1993; Humphreys, 1994). Both result in sodium and water retention and increased interstitial fluid volume presenting as oedema. A detailed review of the pathophysiology of oedema formation is outside the scope of this article, and we direct readers to recent reviews (Siddall and Radhakrishnan, 2012; Cadnapaphornchai et al., 2014; Ray et al., 2015; Ellis, 2016).

## Under-Fill Theory

Hypoalbuminemia reduces the capillary oncotic pressure and the imbalance of Starling’s forces leads to interstitial leakage of fluid and decreased circulating volume (Doucet et al., 2007). The under-fill theory proposes that the decreased circulating volume leads to renal hypoperfusion and activation of the renin-angiotensin-aldosterone system (RAAS) (Perico and Remuzzi, 1993). Stimulation causes avid sodium and water reabsorption (Doucet et al., 2007).

There are data from rat models of nephrosis suggesting that increased activity of the proximal tubular cell sodium/proton antiporter NHE3 may contribute to sodium retention, but this has not been extensively reported (Besse-Eschmann et al., 2002; Klisic et al., 2003). Furthermore, distal delivery of sodium is the same in nephrotic and non-nephrotic kidneys (Ichikawa et al., 1983).

The distal collecting tubule, cortical and outer medullary collecting ducts are the major sites of sodium reabsorption under the control of aldosterone (Mernissi and Doucet, 1983). In nephrosis, cortical collecting ducts demonstrate increased sodium retention associated with stimulation of basolateral Na^+^,K^+^-ATPase and apical epithelial sodium channel (ENaC) (Féraille et al., 1993; Deschênes and Doucet, 2000). The stimulation of Na^+^,K^+^-ATPase results from transcriptional induction of subunits and targeting newly synthesized pumps to the basolateral membrane where the pumps are able to reabsorb sodium (Deschênes et al., 2001a). Pre-formed ENaC is also targeted to the apical membrane via non-transcriptional mechanisms (Lourdel et al., 2005; de Seigneux et al., 2006; Kim et al., 2006).

ENaC is a target for aldosterone and is discussed in detail below. Activation of beta-1 adrenoceptors stimulates renin secretion, however, there are no published studies implicating this in nephrotic syndrome. Renal denervation and beta-blocker therapy are of proven benefit in treating oedema of heart failure (Byku and Mann, 2017), but propranolol did not induce a diuresis or natriuresis (Bauer, 1983) suggesting an alternative unexplained mechanism instead of renin suppression alone.

While the under-fill theory still has considerable support, a sizable body of evidence supports other paradigms of oedema formation. If the under-fill theory was solely responsible then RAAS blockers or restoration of circulating volume would be curative treatment. Captopril (RAAS blocker) failed to change urinary sodium excretion despite successful inhibition of aldosterone secretion (Brown et al., 1984). A proportion of individuals have elevated renin and aldosterone profiles in keeping with an under-filled vascular space, yet, many have suppressed renin and aldosterone activity (Meltzer et al., 1979). Measuring the plasma volume in nephrotic individuals using radioactive albumin demonstrated only 2% of the cohort had a low plasma volume (Geers et al., 1984). Two independent groups in the 1980s demonstrated that intravenous infusion of atrial extract or synthetic atrial natriuretic peptide (ANP) resulted in less than 50% of individuals having a natriuretic and diuretic response despite volume repletion (Koepke and DiBona, 1987; Perico et al., 1989). Interestingly, a low serum albumin alone does not appear to mediate renal sodium retention, as individuals with congenital an-albuminemia do not develop oedema (Koot et al., 2004).

One challenge may be identifying the under-filled individual. A group of Belgian investigators tried to find a clinical test to actively differentiate between these under- and over-filled cohorts (Keenswijk et al., 2018). In nephrotic children a high urinary potassium to urinary potassium and sodium ratio (UrK^+^/UrK^+^ + UrNa^+^), suggesting secondary hyperaldosteronism, can be a useful test to identify under-filled children that may benefit from intravenous therapy (Keenswijk et al., 2018).

## Over-Fill Theory

The over-fill theory states that RAAS activation and imbalance in Starling’s forces across the capillaries are an insufficient mechanism to produce oedema and instead, changes in the capillary endothelial filtration barrier are responsible (Doucet et al., 2007).

## Capillary Permeability

Capillary permeability is important in determining distribution of fluid between vascular and interstitial compartments. Changes in the capillary basement membrane akin to aging exist in nephrotic syndrome, with thickening of the basement membrane, altered protein composition and increased stiffness; the thickening of the basement membrane increases protein permeability (Kottke and Walters, 2016).

Nephrotic patients had higher calf capillary filtration capacity without evidence of capillary hypertension suggesting that the functional capillary surface area available for exchange is increased in nephrotic syndrome (Lewis et al., 1998). The capillary filtration capacity is increased almost twofold in nephrotic individuals (Ellis, 2016).

Studies in nephrotic rats have demonstrated defective capillary basement membrane permeability by ferritin accumulation (Farquhar and Palade, 1961). Vascular permeability is increased in guinea pigs when injected with human lymphocyte supernatants from nephrotic patients but not healthy controls (Lagrue et al., 1975b). A permeability increasing factor (such as a circulating lymphokine) was proposed to affect the vasculature irrespective of the albumin or sodium (Lagrue et al., 1975b). It was hypothesized that this permeability factor was a protein that activates the kinin system (Lagrue et al., 1975a). Initial interest in bradykinin was supported by elevated bradykinin levels in abdominal transudates from nephrotic patients (Paskhina et al., 1979) but no further evidence has been published.

The role for vascular hyperpermeability was further demonstrated using technetium labeled albumin in nephrotic human patients and healthy controls (Rostoker et al., 2000). Nephrotic patients had high levels of hyperpermeability which was reversible with steroids and bilbao extract (which reduces capillary permeability) and the authors proposed a vascular permeability factor derived from local immune cells (Rostoker et al., 2000). The search for the permeability factor remains elusive although possible targets are being proposed such as cytokine receptor-like factor 1 with specific therapeutic targets (Savin et al., 2017). Vascular endothelial growth factors (VEGF) are regulators of capillary permeability (Bates, 2010). However, circulating VEGF levels are not different in nephrotic and non-nephrotic children, and rat VEGF expression is not altered, nor does administration of VEGF induce nephrosis (Webb et al., 1999).

Nephrotic ascites (peritoneal fluid accumulation) is likely to develop via similar mechanisms as nephrotic interstitial fluid (Udwan et al., 2016). In nephrotic rats there is a change in capillary permeability with an increased water filtration coefficient in both paracellular and transcellular pathways, with a reduction in the coefficient for proteins (Udwan et al., 2016). This is associated with greater expression of aquaporin-1 (AQP1) in the parietal peritoneum (Udwan et al., 2016). Water permeability is reversible by inhibiting NF-κB and N-acetylcysteine (Udwan et al., 2016). When both are inhibited, the volume of ascites is reduced by 60% suggesting that this water filtration coefficient, possibly mediated by AQP1, is important in ascites formation in these nephrotic animals (Udwan et al., 2016). The association with aquaporin dysregulation has also been demonstrated in nephrotic human kidneys. A study of 54 primary nephrotic individuals determined aquaporin expression by immunohistochemistry in renal biopsy tissue and measured urinary aquaporin. In nephrotics, AQP1 expression was significantly reduced in renal tissue whereas aquaporin-2 (AQP2) expression was increased. Urinary AQP2 was higher in nephrotic individuals, matching histological findings (Wang et al., 2015).

## Endocrine Effects

Adrenalectomized, aldosterone-deficient rats with puromycin aminonucleoside (PAN)-inducible nephrotic syndrome with ascites have lower levels of apically expressed ENaC in the collecting duct than non-adrenalectomized controls (de Seigneux et al., 2006). This suggests that oedema and nephrotic syndrome can occur in the absence of aldosterone and aldosterone-dependent apical expression of ENaC. Other endocrine and paracrine candidates have been investigated in nephrotic animals to explain aldosterone-independent sodium retention in nephrosis. Inhibition of insulin like growth factor, tumor necrosis factor-alpha, nitric oxide synthase in rat models did not induce natriuresis, implying that these are not involved (Doucet et al., 2007).

Angiotensin II is known to have a direct effect on sodium retention independent of glomerular filtration rate and aldosterone secretion (Johnson and Malvin, 1977; Miura et al., 2014). Microperfusion of angiotensin II in distal nephron segments of rat kidney stimulated transporter-like and channel-like sodium retention (Wang and Giebisch, 1996).

The role of circulating factors on sodium retention are difficult to predict as models of animal unilateral nephrosis demonstrate only alterations in sodium handling in the affected kidney (Doucet et al., 2007). The main determinant of sodium retention is the Na^+^,K^+^-ATPase pump (discussed above) and the capillary epithelial dysfunction.

Atrial natriuretic peptide stimulates sodium and water excretion from the inner medullary collecting duct thereby opposing the interstitial volume expansion (Baxter et al., 1988; Light et al., 1989). After prolonged exposure to ANP there is an increase in apical expression of aquaporin-2 and the gamma subunit of ENaC (Wang et al., 2006). However, systemic infusion of synthetic ANP or ANP extract in experimental studies in nephrotic individuals had a diminished natriuretic and diuretic response compared to healthy controls (Koepke and DiBona, 1987; Perico et al., 1989) despite the prediction from animal models for the opposite (Wang et al., 2006).

Regardless of elevated serum ANP concentration in nephrotic syndrome, natriuresis is blunted (Perico et al., 1989). This may be due to increased activity of renal sympathetic nerves that override the ANP response and stimulate sodium retention, although denervated kidneys still demonstrate the same effect (Maack, 1980). Alternatively, dysfunctional ANP binding in the kidney may explain the relative renal resistance to ANP. In a murine model of nephrotic syndrome, renal resistance to ANP is reversed by phosphodiesterase inhibitors, implying that this ANP resistance is mediated through enhanced cyclic GMP–phosphodiesterase activity (Valentin et al., 1992).

The *PPIL* gene encoding cyclophilin-like protein is upregulated in the medulla from nephrotic rats compared with healthy control rat medullas (Orisio et al., 1993). The gene product cyclophilin-like protein reduces sodium excretion (Iwai and Inagami, 1990). ANP infusion increases the cyclophilin-like protein mRNA in the renal medulla in nephrotic rats, so this may be a potential mechanism of ANP insensitivity in nephrosis (Orisio et al., 1993). Down-regulation of the protease corin in kidneys reduces conversion of pro-ANP to active ANP, also contributing to a lack of renal response to ANP (Polzin et al., 2010).

## Epithelial Sodium Channel

The ENaC mediates absorption of sodium in the late distal convoluted tubule, connecting segment and tubule and the collecting duct (Garty and Palmer, 1997). ENaC is activated by aldosterone, anti-diuretic hormone and specific proteases (Garty and Palmer, 1997; Kleyman et al., 2009; Rossier and Stutts, 2009; Passero et al., 2010). The role of peroxisome proliferator activated receptors oedema and ENaC remains unclear and is reviewed in Pavlov et al. (2010). ENaC is composed of three subunits: alpha, beta and gamma (Firsov et al., 1998). Proteases activate ENaC by cleaving the alpha and gamma subunits (Hughey et al., 2003). Dual cleavage of the gamma subunit renders ENaC highly active (Sheng et al., 2006). Further, animal and human nephrotic urine activates ENaC; proteases (e.g., plasmin) in the urine activate and inhibitors of plasmin deactivate ENaC dependent sodium currents in *Xenopus laevis* oocytes (Svenningsen et al., 2009). Furthermore, remission of nephrotic syndrome is associated with a reduction in urinary plasmin levels in patients’ urine. Patients’ urine was applied to M1 collecting duct cells expressing ENaC and there was a lower ability to activate ENaC-mediated (amiloride sensitive) sodium currents compared to urine from nephrotic patients (Andersen et al., 2013).

Most recently, a cohort study reports urinary plasmin increases the risk of hypertension in type 1 diabetics. Despite this association, the increased risk was dependent on albuminuria and nor were there differences in urinary sodium or potassium excretion. This was attributed to uncontrolled patient sodium intake and urinary plasminogen deactivating ENaC simultaneously (Ray et al., 2018).

Recent studies in mice using pharmacological inhibitors of protease activity to inhibit the gamma subunit cleavage and activation of ENaC have been successful at preventing sodium retention. Urinary protease inhibitors normalized protease activity and reduced sodium retention (Bohnert et al., 2018). Rats with induced nephrotic syndrome had higher levels of urinary plasminogen activator, and amiloride reduced this and sodium retention without altering proteinuria (Stæhr et al., 2015).

Protease inhibition might be an attractive therapeutic option in addition to ENaC antagonism (e.g., with amiloride). Protease inhibition would occur before glomerular filtration, and therefore its effectiveness would not be limited by the glomerular filtration rate, unlike diuretics which act on the tubular epithelium after filtration. Further, while the onset of action of a putative protease inhibitor will be slower than ENaC antagonism, as it depends on the rate of ENaC retrieval to the apical membrane (Gaillard et al., 2010), the effect is likely to be sustained rather than the short action of ENaC antagonism. Avid sodium retention in between periods of action of diuretics has long been observed (Wilcox et al., 1983; Loon et al., 1989), and is an important contributor to diuretic resistance. Protease inhibition may therefore present an intriguing route to ameliorate diuretic resistance in nephrotic syndrome.

In animal models of nephrotic syndrome amiloride successfully abolishes the abnormally high sodium reabsorption from the cortical collecting duct independent of aldosterone activity (Deschênes et al., 2001b, 2003). ENaC inhibitors (amiloride) are not without side effects; they increase serum potassium (Brown et al., 2016) and pose a risk of hyperkalemia so should be used with caution in advanced chronic kidney disease (Wile, 2012) or diabetic patients (Unruh et al., 2017). When used in combination with other medications such as RAAS blockers and diuretics the risk of acute kidney injury is higher (Unruh et al., 2017; Hinrichs et al., 2018; Ray, 2018). There is also an association with pressure ulcers in hospitalized patients on amiloride (Roustit et al., 2016). There are other potential downstream effects of amiloride. Amiloride inhibits urokinase plasminogen activator and reduces plasmin generation (Vassalli and Belin, 1987). Plasmin is pro-fibrotic (Zhang et al., 2007) and therefore amiloride may potentially affect renal fibrosis. Additionally, podocyte anchoring to the glomerular basement membrane is affected by amiloride in experimental animal studies and thereby could influence proteinuria (Zhang et al., 2012; Reiser, 2013; Trimarchi et al., 2014; Warnock, 2015).

Epithelial sodium channel knockout mice exhibited downregulation of the sodium chloride cotransporter (NCC) which was then unable to compensate with the usual enhanced compensatory sodium reabsorption (Perrier et al., 2016). NCC can be phosphorylated and regulated by the serum potassium concentration (Czogalla et al., 2016; Terker et al., 2016). Reduced ENaC activity induces hyperkalemia which may be a more important regulator of NCC than sodium balance (Boscardin et al., 2018). It is possible that amiloride may mediate a degree of NCC inhibition by increasing the serum potassium.

Combination treatments with amiloride have been trialed. Amiloride causes a significant diuresis in mouse models treated with acetazolamide (Patel-Chamberlin et al., 2016). Amiloride and hydrochlorothiazide improved weight loss compared to placebo in a population aged over 65 (Damian et al., 2016). The addition of amiloride to RAAS blockers and hydrochlorothiazide, improved proteinuria reduction by an additional 14% (Morales et al., 2015). Despite the growing evidence for ENaC inhibition, loop diuretics remain the mainstay of treatment.

We have focused on the putative mechanisms of salt and water retention in nephrotic syndrome; however, mechanisms other than renal sodium retention may be important in causing interstitial oedema.

## Treatment of Oedema

### Diuretics

We refer the reader to the review of Wile for details on the history of diuretics (Wile, 2012). All classes of diuretics act within the kidney to reduce renal tubular sodium reabsorption, limiting water reabsorption with resulting diuresis. Diuretics are classed as osmotic diuretics, carbonic anhydrase inhibitors, loop, thiazide or potassium-sparing diuretics (The National Institute for Health and Care [NICE], 2018). All diuretics (with the exception of mineralocorticoid receptor antagonists) act on the luminal side of the tubular epithelium and need to attain sufficient concentration to have an action there (Wile, 2012).

Loop diuretics (e.g., furosemide and bumetanide) work by inhibiting the Na^+^-K^+^-2Cl^-^ cotransporter, NKCC2 on the apical surface in the thick ascending limb (TAL) in the loop of Henle (Brater, 1991). This transporter reabsorbs sodium (Na^+^) in to the tubular cell which is reclaimed to the circulation by the Na^+^,K^+^-ATPase pump in the basolateral membrane (Brater, 1991). The TAL is the site of approximately 25% of total sodium reabsorption in the nephron, so loop diuretics are particularly potent natriuretics (Burg, 1982).

Loop diuretics are highly protein bound and are secreted in to the lumen by organic anion transporters along the proximal tubule to reach their site of action in the TAL (Brater, 1991). Gut oedema may limit the oral absorption of diuretics and hypoalbuminemia decreases the delivery of diuretic to its site of action (Sica, 2003). For this reason higher doses may be required to achieve a successful diuresis, although with a higher likelihood of adverse effects (Crew et al., 2004). Intravenous infusion is another approach that can increase loop diuretic efficacy as therapeutic levels are achieved rapidly due to lower absorption time (Hammarlund et al., 1984).

Furosemide has incomplete and variable bioavailability even in healthy individuals (Waller et al., 1981); probably the worst in its class, despite its widespread popularity. Furosemide has both poor aqueous solubility and low intestinal permeability, and oral furosemide bioavailability varies greatly (by an estimated 20–60%) both between individuals and within individuals (Granero et al., 2010; Nielsen et al., 2016). In the oedematous state bioavailability is further reduced (e.g., to 30%) (Odlind and Beermann, 1980). There is current work using nanoparticles or polymeric microcontainers aiming to stabilize and reduce this effect (Sahu and Das, 2014; Nielsen et al., 2016).

Any diuretics can cause hypovolemia with secondary acute kidney injury (Crew et al., 2004; Oh and Han, 2015). Electrolyte dysregulation is frequent; including hypokalemia, hypomagnesemia, hypocalcemia, hyponatremia, and hyperuricemia. This requires monitoring. Moreover, furosemide can cause hypersensitivity reactions such as skin rashes or acute interstitial nephritis (Oh and Han, 2015). There is a risk of reversible ototoxicity related to the peak serum drug concentration due to the ubiquitous NKCC1 present in the inner ear also being inhibited with loop diuretic use (Wile, 2012). Diuretics can displace warfarin from its protein binding site, increasing the anti-coagulant effect (Oh and Han, 2015). This is seen with furosemide (Oh and Han, 2015) and the active metabolite (canrenone) of spironolactone (Takamura et al., 1997).

### Novel Agents

Vasopressin receptor antagonists (e.g., tolvaptan) are not diuretics, but rather aquaretics. These drugs reduce the density of luminal aquaporins to increase urinary water excretion without natriuresis (The National Institute for Health and Care [NICE], 2018). To date there are three case reports on four individuals describing the use of tolvaptan in massive oedema in nephrotic individuals, with treatment described as successful in three out of four cases (Shimizu et al., 2014; Park et al., 2015; Tanaka et al., 2017).

Phase I trials have been completed for a novel particulate-guanylyl-cyclase A receptor activator (trial name ZD100) that promotes natriuresis, inhibits aldosterone and reduced blood pressure with activation of cyclic guanosine monophosphate (Chen et al., 2016). At present this is being investigated in the use of resistant hypertension but if it has natriuretic properties it may be suitable for use in oedema in nephrosis.

Relaxin, an endogenous neurohormone is currently being trialed in heart failure and has completed phase III trials (Wilson et al., 2015). Relaxin induces increased expression of both epithelial and endothelial endothelin B receptor (ET_B_) and thereby indirectly stimulates ET_B_ (Garvin and Sanders, 1991; Danielson et al., 2000; Bogzil et al., 2005; Schneider et al., 2007). ET_B_ inhibits Na^+^,K^+^-ATPase and ADH causing both a natriuresis and diuresis (Garvin and Sanders, 1991). The fractional increase in urinary excretion of sodium occurs without any changes in aldosterone or ANP concentrations (Bogzil et al., 2005). In heart failure, treated individuals were found to require lower doses of loop diuretics, had greater weight loss and reduced signs and symptoms of fluid overload including peripheral and pulmonary oedema (Metra et al., 2013; Voors et al., 2014). However, this has had no effect on overall outcomes (Teerlink, 2017; Teerlink et al., 2017), and nor is it known if the endothelin dysregulation in heart failure is present in nephrosis.

There have been two cases from a single center in Japan of synthetic human ANP (carperitide) reducing interstitial oedema preventing the need for hemodialysis and preserving renal function in nephrotic patients (Ueda et al., 2014).

Luteolin is a common phenolic compound known to have anti-inflammatory and anti-allergic effects. Rat studies have recently demonstrated the role of luteolin in natriuresis and diuresis with an additive effect achieved with administration of amiloride and hydrochlorothiazide (Boeing et al., 2017). Luteolin mediated these effects via the muscarinic acetylcholine receptor (Boeing et al., 2017). It will be interesting to see if this has a similar role to play in humans.

Most recently, the role of epicatechin was investigated. Epicatechin is a flavonoid found in food and plant extracts and is classed as a phytochemical (Mariano et al., 2018). Rats treated with epicatechin achieved diuresis and uresis of sodium, potassium and chloride without any effect on the plasma electrolyte function (Mariano et al., 2018). Combination with hydrochlorothiazide further improved the diuretic effect (Mariano et al., 2018).

### Clinical Practice

There are no adult guidelines available on managing oedema and volume overload in nephrotic syndrome (Crew et al., 2004; Oh and Han, 2015). The lack of guidelines means that there is considerable heterogeneity in the treatment of overloaded nephrotic individuals with no clear consensus. Non-pharmacological interventions such as strict dietary sodium restriction (less than 3g per day) are important and have an additive effect to other therapies (Hull and Goldsmith, 2008). Replacing serum albumin with intravenous infusions to improve the efficacy of loop diuretics has been investigated considerably. This is because only albumin-bound loop diuretics are secreted into the lumen of the tubule, discussed above. Hypoalbuminemia (as is present in nephrotic syndrome) reduces the amount of loop diuretics that are delivered to their site of action in the tubular lumen (Kirchner et al., 1990; Fliser et al., 1999). Albumin may help overcome this by enhancing proximal tubular secretion to the tubular lumen and reducing the volume of distribution (Inoue et al., 1987; Chalasani et al., 2001). Early reports were encouraging with massive diuresis when combined with diuretics (27 kg in 14 days) (Davison et al., 1974). Later studies demonstrate conflicting results with better results in animal models and minimal response in humans. There was little or no effect on urinary furosemide or sodium excretion, despite elevated serum albumin levels (Akcicek et al., 1995; Fliser et al., 1999; Chalasani et al., 2001). Nor did inhibiting urinary protein binding have any effect of furosemide efficacy (Agarwal et al., 2000). Different mechanisms may exist in children as there has been a successful response with co-administration of albumin in some instances (Haws and Baum, 1993). Yet, the rate of complications remains higher in pediatric patients with 38% developing hypertension (Dorhout Mees, 1996). We refer the reader to table 1 in the following review for a full synopsis on all clinical trials with albumin and furosemide (Duffy et al., 2015).

A multicenter retrospective study of 60 pediatric units in Italy found no consensus approach to diuretic treatment (Pasini et al., 2015). Despite similar laboratory and clinical data across 231 children, 64% were treated with diuretics and 55% were treated with albumin infusions, highlighting the treatment variability (Pasini et al., 2015). The authors suggest that shared guidelines and implementation were necessary to avoid differences and side effects in pediatric patients (Pasini et al., 2015).

In adults, there is also no consensus on the indication, starting dose, approach to dosage change and monitoring of diuretics; consequently, there are considerable differences in treatment pathways. In general, standard first line treatment is a loop diuretic such as furosemide (Crew et al., 2004). The diuretic would normally be started at a low dose and then sequentially increased until satisfactory weight loss or until the maximum dose has been reached. If at this stage the patient remains symptomatic, a second, synergetic diuretic is commonly added. In the United Kingdom, this would usually be the thiazide-like metolazone (Crew et al., 2004). This is not always successful and a recent case report described resistance to the loop and thiazide type diuretic combination in a oedematous nephrotic patient. The oedema instead responded well to a combination of furosemide and an ENaC inhibitor (triamterene) (Hoorn and Ellison, 2017). Another case report describes a treatment resistant nephrotic patient with no diuretic effect after 5 weeks of high dose furosemide but a profound 7 kg fluid loss with the addition of amiloride (Hinrichs et al., 2018). While these are only two case reports they provide further evidence to support ENaC blockade as effective treatment of nephrotic oedema, although larger studies will be necessary.

There are no trials directly comparing the commonly used different diuretic regimens in nephrotic syndrome despite the underlying scientific basis of causation for oedema in animal models. Furosemide remains first line treatment yet has extremely variable oral bioavailability and the problematic well-described blunted natriuretic effect with prolonged therapy (Bernstein and Ellison, 2011).

More recently, a randomized controlled study in Iran compared different diuretic pre-loading regimens in 20 individuals with refractory nephrosis. The two arms were either pre-loading with acetazolamide and hydrochlorothiazide compared to pre-loading with furosemide and hydrochlorothiazide for 1 week after which both treatment arms received 2 weeks of 40 mg furosemide. Patients with hypokalemia were excluded in view of the potassium wasting properties of these diuretics. The authors concluded that the acetazolamide and hydrochlorothiazide combination achieved a better diuresis. While there was a difference in the mean weight change and urinary volume there was no difference in the urinary sodium and hence natriuresis over the trial period (Fallahzadeh et al., 2017). We discussed above that this was successful in animal models (Patel-Chamberlin et al., 2016). Inhibition of pendrin which is found in the collecting duct is part of a proposed route of sodium reabsorption in the collecting duct with acetazolamide (Zahedi et al., 2013). This may be another mechanism for the synergistic effect with a loop diuretic (Soleimani et al., 2012).

## Conclusion

Patients and clinicians deserve better and more structured information on how to successfully manage nephrotic oedema. With increased understanding of the underlying pathophysiology of interstitial oedema in nephrotic syndrome we are in a better position to better treat individuals suffering with the complications that severe interstitial oedema brings. Management of oedema in nephrotic syndrome will rely on individualization of therapy with consideration of the clinical evidence. Differences especially will exist in the treatment between pediatric and adult patients and it is likely that there is no one best treatment for everyone. It will be helpful to understand determinants of response to treatment. Based on the evidence we provide in the review we especially advocate clinical trials to investigate the potential benefits of ENaC blockade. For example, it may be important in certain types of drug-induced oedema, such as that seen with thiazolidinediones,which stimulate ENaC-mediated sodium reabsorption through PPARγ receptors (Guan et al., 2005). We hope that with appropriately designed trials, guidelines can be developed based on robust clinical evidence to achieve improved outcomes.

## Author Contributions

SG drafted the manuscript with critical revision by RJP, NA, and SBW.

## Conflict of Interest Statement

The authors declare that the research was conducted in the absence of any commercial or financial relationships that could be construed as a potential conflict of interest.
